# Continuous Motion Estimation of Knee Joint Based on a Parameter Self-Updating Mechanism Model

**DOI:** 10.3390/bioengineering10091028

**Published:** 2023-08-31

**Authors:** Jiayi Li, Kexiang Li, Jianhua Zhang, Jian Cao

**Affiliations:** 1 School of Mechanical Engineering, Hebei University of Technology, Tianjin 300401, China; 201911201002@stu.hebut.edu.cn (J.L.); 202111201001@stu.hebut.edu.cn (J.C.); 2 School of Mechanical and Materials Engineering, North China University of Technology, Beijing 100144, China; likex_2023@ncut.edu.cn; 3 School of Mechanical Engineering, University of Science and Technology Beijing, Beijing 100083, China

**Keywords:** continuous joint motion estimation, surface electromyography (sEMG), deep learning, feature extraction, particle swarm optimization (PSO), deep belief network (DBN)

## Abstract

Estimation of continuous motion of human joints using surface electromyography (sEMG) signals has a critical part to play in intelligent rehabilitation. Traditional methods always use sEMG signals as inputs to build regression or biomechanical models to estimate continuous joint motion variables. However, it is challenging to accurately estimate continuous joint motion in new subjects due to the non-stationarity and individual differences in sEMG signals, which greatly limits the generalisability of the method. In this paper, a continuous motion estimation model for the human knee joint with a parameter self-updating mechanism based on the fusion of particle swarm optimization (PSO) and deep belief network (DBN) is proposed. According to the original sEMG signals of different subjects, the method adaptively optimized the parameters of the DBN model and completed the optimal reconstruction of signal feature structure in high-dimensional space to achieve the optimal estimation of continuous joint motion. Extensive experiments were conducted on knee joint motions. The results suggested that the average root mean square errors (RMSEs) of the proposed method were 9.42° and 7.36°, respectively, which was better than the results obtained by common neural networks. This finding lays a foundation for the human–robot interaction (HRI) of the exoskeleton robots based on the sEMG signals.

## 1. Introduction

As a rehabilitation robot, the lower limb exoskeleton is advantageous in helping elders or disabled people to walk. Accurate estimation of the continuous motion of the knee joint is one of the critical research components for high matching of human–robot coordinated motion. Surface electromyography (sEMG) signals are potentials and signals excited by neurons carrying human behavioural information when transmitted to the relevant tissue/organ, directly reflecting human intention [1]. sEMG signals reflect the neuromuscular activity level to a certain extent, and they are generally generated 30–150 ms ahead of limb movement [2]. The sEMG-based continuous motion estimation methods for the human knee joint are mainly classified into the biomechanical model method and the regression model methods [2].

Biomechanical modelling is usually accomplished by modelling human muscle forces based on the Hill-type muscle model and constructing a geometric model of the human skeleton to achieve continuous joint motion estimation. This method can explain the mechanism of human motion generation, but the model contains many unknown physiological parameters that must be effectively calculated [3,4,5,6]. In contrast to complex biomechanical models, the regression model method usually establishes a direct mapping relationship between the sEMG signals and the continuous motion of the joint, such as back-propagation neural network (BPNN) [7,8,9], multilayer perceptron (MLP) [10], artificial neural network (ANN) [11,12,13], Elman network [14], general regression neural network (GRNN) [15], Adaboost [16], and Gaussian process autoregression [17]. These regression models typically involve the use of the time domain features as inputs. Then, the corresponding model training or polynomial calculation is performed according to the obtained features for the estimation of joint motion variables [2]. Neural-network-based continuous estimation of joint angles (2D) is typically applied in exoskeleton control system applications [18].

However, traditional time domain features, such as absolute mean value (MAV) [9,10,13], root mean square (RMS) [8,11,12,14,15,16], and other artificial selection of features are likely to cause the loss of useful information in the original sEMG signals, which reduces the accuracy and robustness of sEMG recognition. Accordingly, it is necessary to apply deep learning models with high accuracy and robustness [19,20]. Deep learning networks, including deep belief networks [21], long short-term memory (LSTM) [22,23,24,25], recurrent neural networks (RNN) [26], and convolutional neural networks (CNN) [27], are used to perform the self-learning and hierarchical feature representation of sEMG features, thereby improving the accuracy of estimated results. Gautam [23] proposed a long-term recurrent convolutional network (LRCN) based on transfer learning to estimate the knee joint angle, using CNN to extract features directly from the raw sEMG signal and LSTM for sequence prediction. Ma [24] proposed a high-level temporal feature (RMSTAF) incorporating RMS features, and LSTM was used to estimate knee joint angle. Ma [25] proposed a method for elbow joint angle estimation based on the short-connected autoencoder long short-term memory (SCA-LSTM) model. This method adopted an autoencoder to extract the common-mode features of the sEMG signals and combine the short connection to separate the different-mode signals. Wang [27] proposed a CNN that could directly use the raw sEMG signals as input to predict the continuous motion trajectory about the three degrees of freedom (DOF) of the wrist.

Despite the satisfactory results of the current study, the conventional fixation model does not allow better identification of new subjects. Since the sEMG signals are typically non-linearity real-time signals with individual differences, they are closely related to the physical signs of the subjects. Individual differences have gradually become one of the main problems hindering the application of the sEMG interaction system. How to obtain personalized models satisfying different subjects has become a key technology in this area. To solve the above-mentioned issues and improve the estimation accuracy, we developed a model of parameter self-updating mechanism to compensate for the impact of strong non-linearity and individual differences in sEMG signals on estimation accuracy.

The main contributions of this study could be seen as follows:

1. A self-adaptive optimized DBN, depending on the original sEMG signals of different subjects, was built to complete the reconstruction of sEMG sequences.
2. An adaptive regression model fused with BPNN was established to achieve the optimal estimation of continuous joint angle.
3. A parameter self-updating mechanism was applied to update the model parameters using a small amount of data from new subjects to satisfy personalized demand.

## 2. Materials and Methods

**Figure 1:**
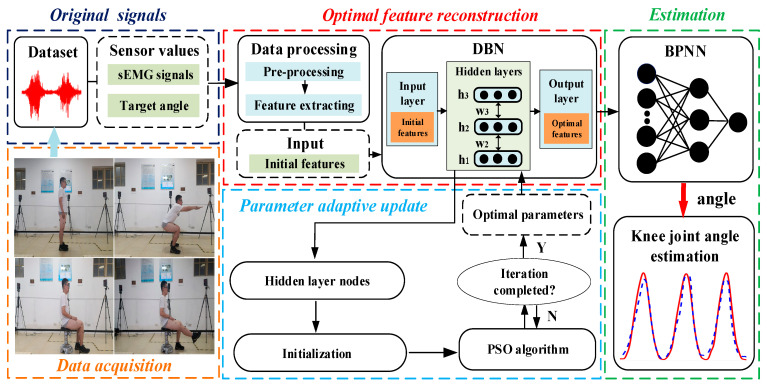
Flow chart of the method.

To obtain personalized models with parameter self-updating mechanisms for different subjects, the methods of acquiring and processing the raw signals were first detailed in this section. Then, the process of initial feature extraction and the feature reconstruction methods were presented. Next, the adaptive regression model was built by integrating BPNN. Finally, we introduced three evaluation indicators of results. The algorithm flow can be found in Figure 1.

### 2.1. Data Acquisition and Pre-Processing

**Figure 2:**
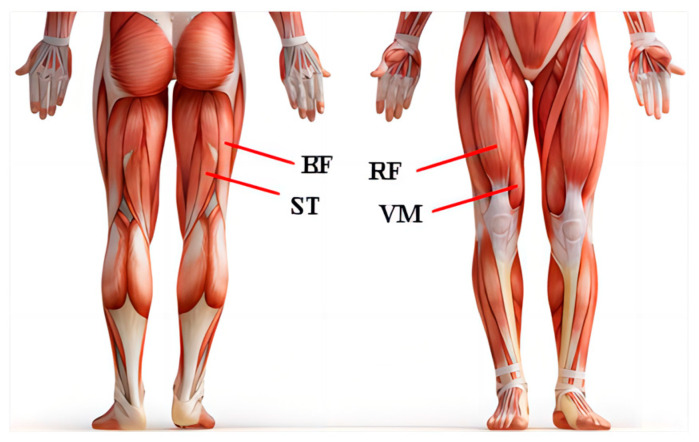
Wireless sEMG sensor electrode locations.

For continuous estimation of the knee joint angle, two to five groups of muscles will normally be selected to obtain sEMG signals [14,17,23,26]. In this study, we selected the Rectus Femoris (RF), Vastus Medius (VM), Biceps Femoris (BF), and Semitendinosus (SE) based on previous research [23]. The RF and VM control knee joint extension [28]. BF and SE dominate the flexing and rotation of the knee joint [29]. Four wireless sEMG sensors (Trigno^TM^, Delsys Corporation, Natick, MA, USA) were used to acquire raw sEMG signals with a frequency of 1928 Hz. The layout scheme is shown in Figure 2. The optimal location for the sensors is on the midline of the muscular abdomen between the nearest innervated area and the tendon junction [30,31,32,33]. At this position, the sEMG signals with the greatest amplitude are obtained [33,34].

We used a 6-camera optical motion capture system (VICON, UK, Oxford Measurement Ltd.) to record the information on lower limb movements. This was a motion capture and analysis device that could use active infrared to obtain object motion information, with a sampling frequency of 100 Hz.

**Figure 3:**
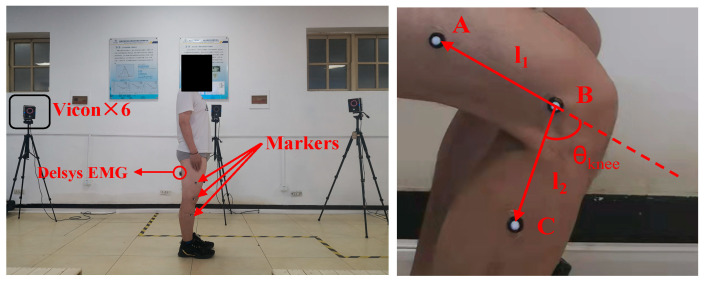
Motion capture system.

We pasted the marking points for all subjects according to the Plug-in Gait model provided in the VICON system. The body dimensions of the subjects should be measured before pasting the marking point, including lower limb length, knee, and ankle breadth. The rotation centre B was determined to be placed on the lateral epicondyle of the right knee. We needed to ensure that marker B on each subject was located at the intersection of the midline of the thigh and shank. Markers A and C were located near the midline of the thigh and shank, respectively [6,14]. To calculate the knee joint angle, we connected the three luminous markers A, B, and C in a simplified 2-DOF linkage, as shown in Figure 3. The coordinate positions of A, B, and C are defined as *P_A_* = [*x_A_*, *y_A_*, *z_A_*], *P_B_* = [*x_B_*, *y_B_*, *z_B_*], *P_C_* = [*x_C_*, *y_C_*, *z_C_*]. Therefore, the formula for calculating the angle of the knee joint is:

(1) $$\theta_{knee}=180^{\circ }-\arccos\left(\frac{\vec{l_{1}}\cdot \vec{l_{2}}}{\left|l_{1}\right|\left|l_{2}\right|}\right)$$

where *θ_knee_* is the angle between the extension line of the thigh chain *l*_1_ and the shank chain *l*_2_, *l*_1_ = [*x_A_* − *x_B_*, *y_A_* − *y_B_*, *z_A_* − *z_B_*], *l*_2_ = [*x_C_* − *x_B_*, *y_C_* − *y_B_*, *z_C_* − *z_B_*].

We used the kinematic information extracted from the sEMG signals to estimate the continuous knee joint angle, and the actual values calculated by the VICON system served as data labels to verify the estimation.

sEMG signals are nonlinear, and their effective frequency is mainly distributed at 10–500 Hz. The raw sEMG signals are doped with many interference signals. The interference signals mainly include the inherent noise in electronic instruments distributed from 0 to several kHz and power frequency interference with specific frequencies of 50 or 60 Hz in power systems [35]. All interfering signals should be removed. As per the above characteristics of the interfering signals, a 10–400 Hz bandpass filter and a 50 Hz trap filter (with a bandwidth of 2 Hz) were applied to remove the interfering signals.

After eliminating the interference, the signals were full-wave rectified and segmented using the overlapped window technique [36]. Overlapped windowing techniques use the idle time of the processor to generate more classified outputs [35]. The window length should generally be longer than 125 ms so that it does not lead to high variance and bias in features [37,38]. In this study, the duration of the analysis window was 180 ms, and the overlapping was 80 ms. Therefore, the root mean square (RMS) was adopted in this work to extract the initial features:

(2) $$RMS=\sqrt{\frac{1}{N}\sum_{i=1}^{N}sEMG{\left(i\right)}^{2}}$$

where *N* is the number of sampling points. *sEMG*(*i*) represents the *i*th sampling. At the same time, we used the analysis window to process the angle value [8], as shown in Equation (3):

(3) $$\bar{A}=\frac{1}{N}\sum_{i=1}^{N}A_{i}$$

where $\bar{A}$ is the average angle, *A_i_* is the angle value of the *i*th sampling, and *N* signifies the number of sampling points, here *N* = 10.

### 2.2. Feature Reconstruction by DBN

**Figure 4:**
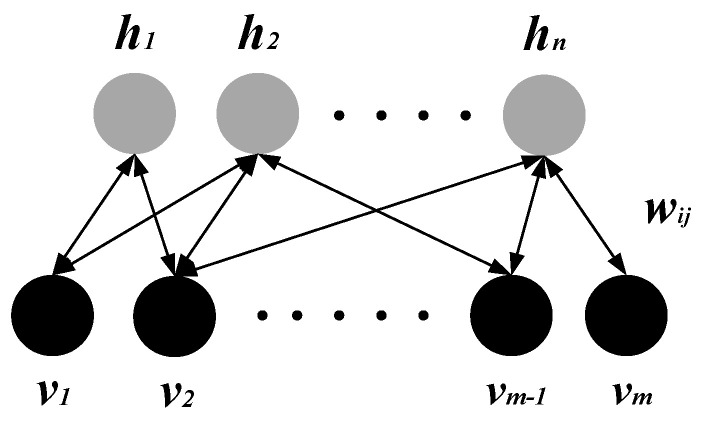
RBM structure.

For autonomous learning of advanced structural contents from strong nonlinear datasets to obtain the optimal features in high-dimensional space, we built a DBN to reconstruct the initial features of different subjects. It was composed of multiple Restricted Boltzmann Machines (RBM) stacks that could be applied to reconstruct the dimensionality of the input vectors. For such a multilayer network, the output of each layer contained all the information of the input data, which could reveal the hidden nonlinear structures in high-dimensional data [39,40]. Figure 4 presents the RBM topology, including the visible and hidden layers. *v* = (*v*_1_, *v*_2_, …, *v_m_*)^T^ represents the normalized multi-dimensional vector of the input layer. *m* is the number of neurons in the input layer. *w_ij_* is the weight matrix. *h* = (*h*_1_, *h*_2_, …, *h_n_*)^T^ denotes the multi-dimensional vector of the hidden layer. *n* is the number of neurons in the hidden layer. The concept and training process of RBM are presented below.

In the binary RBM, the neurons are all Boolean and can only take two states of “0” and “1”, “0” represents the inhibitory state, and “1” represents the activation state. The model parameters determine the energy function between the visible and hidden layers and the energy function is expressed as:

(4) $$E\left(v,h\right)=-\sum_{i=1}^{n}\cdot \sum_{j=1}^{m}W_{ij}h_{i}v_{j}-\sum_{j=1}^{m}b_{j}v_{j}-\sum_{i=1}^{n}c_{i}h_{i}$$

where *w* is the weight matrix between the visible and hidden layer, *b* denotes the bias vector of the visible layer, and *c* refers to the bias vector of the hidden layer.

Based on the energy function, we further obtained the joint probability distribution function of (*v*, *h*):

(5) $$P(v,h)=\frac{e^{-E(v,h)}}{\sum_{v,h}e^{-E(v,h)}}$$

Each neuron in the same layer is independent of each other, so the activation function of the neuron can be obtained by Equations (6) and (7):

(6) $$P\left(h_{j=1}|v\right)=\sigma \left(v^{T}w_{i,j}+b_{j}\right)$$

(7) $$P\left(v_{i=1}|h\right)=\sigma \left(w_{i,j}h+c_{i}\right)$$

The purpose of training the RBM was to find the optimal weight parameters to obtain the optimal solution. The output of the hidden layer should generate training data according to the maximum probability, which could more accurately reflect the characteristics of the input data. The RBM model reached the ideal state when the energy function is the smallest, so the training optimization objective function would be given as follows:

(8) $$\max L\left(\theta \right)=\prod_{v}P\left(v;\theta \right)$$

We selected the Contrastive Divergence (CD) algorithm to solve the model parameters to achieve the fastest and most effective training effect. The updated formulas of weights and biases could be observed as follows:

(9) $$w_{ij}=w_{ij}+\varepsilon \left(\langle v_{i}h_{j}\rangle -\langle v_{i}^{\prime }h_{j}^{\prime }\rangle \right)$$

(10) $$a_{i}^{\prime }=a_{i}+\varepsilon \left(\langle v_{i}\rangle -\langle v_{i}^{\prime }\rangle \right)$$

(11) $$c_{j}^{\prime }=c_{j}+\varepsilon \left(\langle h_{j}\rangle -\langle h_{j}^{\prime }\rangle \right)$$

**Figure 5:**
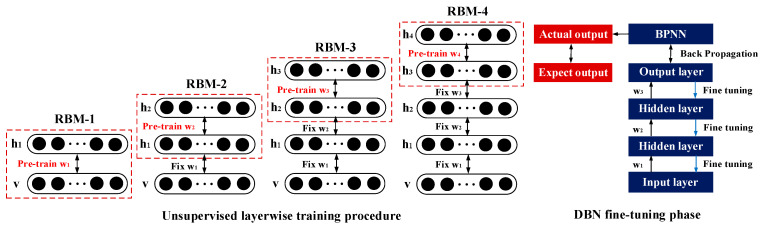
The whole training process of DBN.

The training process of DBN included two steps: pre-training and fine-tuning. Firstly, a stack of RBMs was trained layer-wise. Each layer of RBM was trained separately and unsupervised to ensure that feature information was preserved as much as possible when mapping to different feature spaces. The high-level features were extracted from the lower layers and moved forward layer by layer until the top layer. After pre-training, the RBMs were expanded to obtain the DBN. Secondly, to minimize the error between the expected output of the model and the actual output, the back-propagation algorithm (BP) was adopted to fine-tune the model parameters and as a result, the optimal weight of the structure could be obtained. The loss function could keep the predicted value close to the true value. When the loss value was the lowest, the predicted value and the true value were equal. The whole process is shown in Figure 5.

In this paper, we constructed a self-adaptive optimized DBN, which had four layers to build the optimal reconstruction of initial features. The network parameters were optimized using the PSO algorithm. Then, the RMS features were encoded by PSO-DBN to obtain the optimal reconstructed features. The number of neurons in the visible layer was 4, and the initial feature vectors were used as input data. The higher dimensional outputs of the top level served as the optimal reconstruction features of sEMG. It must be emphasized that the dimension of the reconstructed feature vectors was determined by the number of neurons for the top layer of PSO-DBN.

### 2.3. DBN Adaptive Optimization Fused with the PSO Algorithm

In this study, we investigated the reconstruction of sEMG features of different individuals in high-dimensional space using DBN. Structural parameters of each layer in DBN directly affected the estimation performance. However, in previous studies, these parameters were manually set through experience, resulting in randomness in feature learning [21]. These fixed parameter models have limited adaptability and cannot satisfy the demands of different subjects. The PSO algorithm has powerful and efficient search capabilities, which can easily obtain the optimal solution in the design space. Therefore, according to the original sEMG signals of different subjects, the parameters of the DBN were adaptively optimized by integrating the PSO algorithm to complete the optimal reconstruction of signal feature structure. The basic idea of the PSO algorithm is to search for the global optimal solution through cooperation and competition between particles. We took the number of hidden layer nodes of the DBN as the optimization variable of the particles and calculated the adaptive value of the objective function by constantly updating the speed and position of the particles to achieve global optimization and obtain the optimal network parameters. The update formulas of the PSO algorithm speed and position can be found in Equations (12) and (13).

(12) $$V_{i}^{k+1}=wV_{i}^{k}+c_{1}r\left(pbest_{i}^{k}-X_{i}^{k}\right)+c_{2}r\left(gbest_{i}^{k}-X_{i}^{k}\right)$$

(13) $$X_{i}^{k+1}=X_{i}^{k}+V_{i}^{k}$$

where *w* signifies the weight, *c*_1_ and *c*_2_ denote learning factors, and *r* is a random number between 0 and 1. *V_i_^k^*, *X_i_^k^*, *pbest_i_^k^*, and *gbest_i_^k^* are the velocity, position, local optimum, and global optimum of particle *i* at the *k*th iteration, respectively. The linear decreasing weight method was applied to ensure that the PSO algorithm had a good ability to balance search and development.

(14) $$w=\left(w_{\max}-w_{\min}\right)\left(k_{\max}-k\right)/k_{\max}+w_{\min}$$

where *w*_max_ and *w*_max_ refer to the maximum and minimum values of inertia weights, *k* is the current number of iterations, and *k*_max_ is the maximum number of iterations.

The parameter self-updating mechanism based on the fusion of PSO and DBN is as follows:

Step 1: Set the particle population size, learning factor, maximum speed, and maximum number of iterations, and then randomly assign all particles an initial position and initial speed within the corresponding search domain.

Step 2: Build a DBN framework for feature reconstruction of original sEMG signals. The number of hidden layer neurons as model parameter variables needs to be determined by optimization calculation within a set range. It will generate a population particle and then set a parameter search range.

Step 3: Determine the parameters of the DBN through the iterative calculation to obtain the optimal structure.

Step 4: Set fitness values of different particles to represent the global and local optimal positions of the particles. They are then used as their historical optimal positions and updated to the public and local optimal particle positions.

Step 5: Output the results of a set of optimal parameters to the DBN when the number of iterations is reached. Otherwise, return to Step 4 to continue the iterative calculation. 

### 2.4. Construction of the Adaptive Regression Model

To improve the generalization ability of the model, we introduced an updating mechanism to enable the model to recognize and remember the motion information of new subjects. In this section, we built a three-layer BPNN connected to PSO-DBN to generate the adaptive regression model. The optimal reconstructed features of sEMG were applied as input. The number of input layer nodes in BPNN was equal to the number of top-level nodes in DBN optimized by the PSO algorithm. The output layer had one node, which was the output of an estimated angle. After extensive testing, the hidden layer neurons of BPNN were set to 12 [21]. The nonlinear tansig function and the linear purelin function were, respectively, selected as the transmission functions in the mid-layer and output layer [9]. Therefore, the output of the BP neural network can be calculated by Equation (15):

(15) $$\hat{\theta }=W_{out}\left[\frac{2}{1+e^{-2\left(W_{in}y+b_{in}\right)}}-1\right]+b_{out}$$

where *W_in_* represents the weight matrix of the hidden layer, the *W_out_* denotes the weight matrix of the hidden layer, and *b_in_* and *b_out_* are threshold vectors. We used the test sets to verify the results when all the parameters were updated.

### 2.5. Result Evaluation Indicators

Based on the above method, the knee joint angle *θ_knee_* could be estimated using the sEMG signals. This paper specified three metrics to evaluate the quantitative difference between actual and estimated values in all regression models: root mean square error (RMSE), correlation coefficient (CC), and R^2^ score.

(16) $$RMSE=\sqrt{\frac{1}{N}\sum_{i=1}^{N}\left(\theta_{i}-\overset{\wedge }{\theta_{i}}\right)}$$

(17) $$\rho \theta_{i}=\frac{\mathrm{cov}\left(\theta_{i},\overset{\wedge }{\theta_{i}}\right)}{\sigma_{\theta_{i}}\cdot \sigma_{{\hat{\theta }}_{i}}}$$

(18) $$R^{2}=1-\frac{\sum_{i=1}^{N}{\left(\theta_{i}-{\overset{\wedge }{\theta }}_{i}\right)}^{2}}{\sum_{i=1}^{N}{\left(\theta_{i}-\bar{\theta }\right)}^{2}}$$

where $\theta_{i}$ represents the actual values, $\hat{\theta }$ refers to the estimated values, *N* is the sample size, $\sigma_{\theta_{i}}$ and $\sigma_{{\hat{\theta }}_{i}}$ are the standard deviations, and $\bar{\theta }$ signifies the mean value of estimations.

In addition, two commonly adopted regression models [9,21] were utilized and compared with our method. One-way analysis of variance (ANOVA) was applied to evaluate the statistical differences in the errors of estimation (*RMSE*) obtained by different models [41]. The statistical significance level was set to *p* < 0.05.

## 3. Experiments and Results

### 3.1. Subjects

Five healthy subjects (male, age: 27 ± 3 years, height: 168–180 cm, bodyweight: 65–85 kg) participated in this experimental study [3,4,7,8,42]. They gave their informed consent before the experiments. The experimental protocol was reviewed and approved by the Ethical Review Committee of the Hebei University of Technology. All subjects had no disorder in range of motion and did not participate in strenuous exercise before the experiment.

### 3.2. Experimental Procedure

In this study, we selected two typical continuous knee motions for this experiment: squat and knee flexion–extension (flex/ext) [6,10,14,17,23]; these motions are commonly used in lower limb rehabilitation training. Before applying the electrodes, the subject’s skin surface was shaved, and the corresponding patch locations were wiped and cleaned with alcohol. With the subject standing upright and arms raised horizontally, the sEMG sensors and luminous markers were attached to the subject’s right leg.

**Figure 6:**
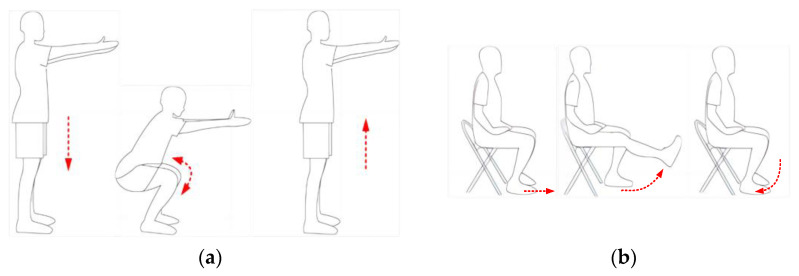
Experimental process of two motions: (**a**) squat; (**b**) knee flexion/extension.

During the preparation stage, the observer accurately recorded the physiological information (age, height, and weight) of each subject and described the experimental process to them. After all the equipment was functioning properly, the subjects performed squatting and knee flexion–extension exercises in shorts as previously required, as shown in Figure 6. During squatting, subjects were asked to stand up and squat at normal speed with their feet shoulder-width apart and toes pointed outward by approximately 15°. After squatting, they kept their thighs parallel to the ground and returned to the preparatory position. It was required that the vertical line from the knee to the floor not extend beyond the toes [10]. Subjects were asked to sit and maintain the knee joint in a constant position at 90° in the knee flex/ext. The amplitude of the movement was limited to the range of 0° (flexion) to 90° (extension) [6,14]. The two motions were limited to the sagittal plane.

Each subject performed 20 repetitions continuously at the same speed for each of the two motions. It resulted in a total data set of 5 subjects × 20 repetitions × 2 modes. A metronome was adopted to guide the movement of the knee joint. The subjects completed half a cycle per beat. The wireless sEMG sensors and vision system simultaneously acquired raw sEMG signals and “true” angle values.

The interval between each trial was 5 min to avoid muscle fatigue [8,10,14]. During each trial, if subjects felt muscle fatigue, they were told to stop the trial immediately. Meanwhile, the recorder observed real-time changes in the median frequency (MF) of the four muscles. The MF of spectrum analysis after fatigue would be reduced by more than 50%. This test ensured that all data were obtained in a state without fatigue. The duration of each trial was approximately 80 s.

In this work, the experimental data of each subject were divided into two parts: training set and testing set. The percentages of the training data for one subject were set to 70%. The remaining 30% of one subject’s data was used as testing data. We calculated the RMS features of the four sEMG channels. Finally, the RMS features were formed into four-dimensional initial feature vectors.

### 3.3. Model Training

We built separate regression models for each subject and the model was trained with the training set, including the sEMG initial feature vectors *v* = [*v*_1_, *v*_2_, *v*_3_, *v*_4_]^T^ and the “real” angle data *θ_knee_*.

**Figure 7:**
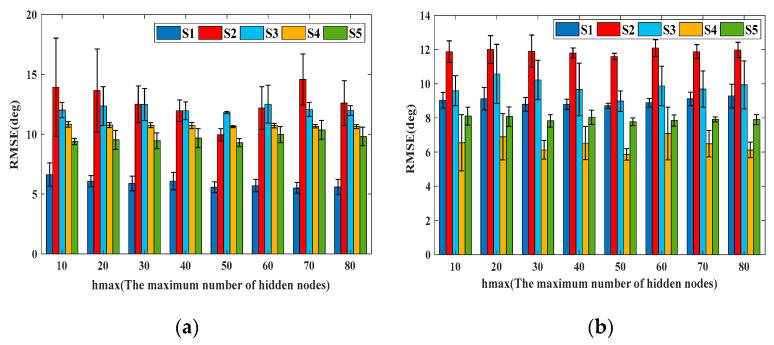
(**a**) The RMSEs of squat with respect to different ranges of hidden nodes; (**b**) the RMSEs of knee flex/ext with respect to different ranges of hidden nodes.

The parameters of the DBN were optimized as per the PSO algorithm, as required: the number of particle populations *N* = 20, the total number of iterations *i*_max_ = 10, the learning factors *C*_1_ and *C*_2_ were 0.9 and 0.5, the random number was 1, *w*_max_ and *w*_min_ were 0.9 and 0.5, respectively. The number of hidden layer nodes in DBN had to be considered for the performance and the computational cost of network training. The minimum error and the lowest computational load parameters were chosen. We explored the impact of the range of hidden layer nodes on the estimation results. Figure 7 shows the RMSEs under different ranges of hidden layer nodes. The number of hidden layer nodes was between 1 and the maximum number of hidden layer nodes (hmax). The best performance was achieved when hmax was set to 50. However, when selecting other hmax values, relatively large errors would present due to the network not achieving optimal performance.

**Figure 8:**
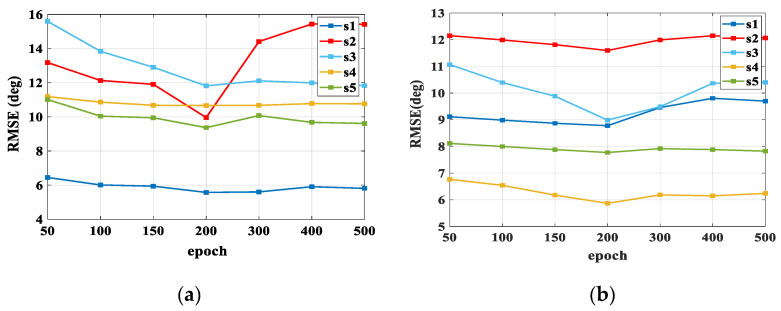
(**a**) The RMSEs for PSO-DBN trained by different epochs in squat; (**b**) the RMSEs for PSO-DBN trained by different epochs in knee flex/ext.

After the parameter optimization, we used the method mentioned in 2.4 to train the DBN. The first step was to perform hierarchical pre-training on RBMs. The initial feature vectors *v* were used as input data to train the RBM-1. After reaching the epoch number, we fixed the weight of RBM-1 and used its output to train RBM-2. We then trained layer by layer until the end. The learning rate of the RBM was 0.01. After one iteration, we could update the weights over all the training data, but batching the training set would be more efficient. After training the RBMs, we expanded the RBMs to the DBN with the same model parameters. The BP algorithm could effectively fine-tune the parameters. Figure 8 shows the RMSEs of using different numbers of epochs. It was observed that when the number of epochs reached 200, the accuracy was the highest. The results of the parameter optimization and training time for all subjects are summarized in Table 1.

After the DBN training was completed, the optimal features from the PSO-DBN reconstruction were applied as the input of the BPNN. The “true value” *θ_knee_* measured using Vicon is used as the training output. The maximum number of iterations is 1000, and the learning rate is 0.01. 

**Figure 9:**
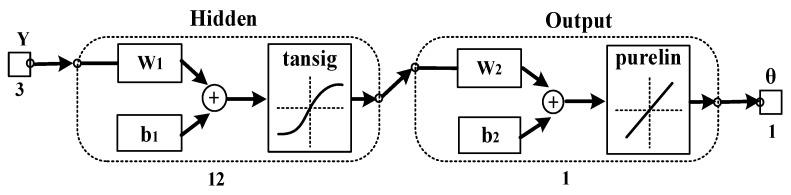
Training process of the BPNN connected to DBN with fixed parameters.

**Figure 10:**
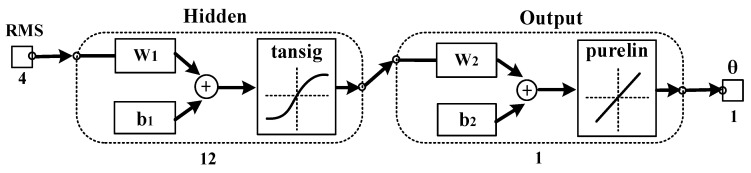
The training process of the normal BPNN.

We also built a DBN with fixed parameters and a normal feed-forward BPNN model for performance comparison. To make the comparison results more convincing, we set the numbers of nodes for each layer of DBN with fixed parameters as 18, 12, 6, and 3, which were the same as [21]. The input of the DBN with fixed parameters was the four-dimensional sEMG initial feature vectors and the actual angle values *θ_knee_* were the output. The number of BPNN input layer nodes was 3. Figure 9 illustrates the trained BPNN structure. *Y* is the input sEMG feature vectors, *θ* signifies the output estimate angle, and *W*, *b* represents the weight and bias, respectively. The numbers of neurons are 3, 12, and 1. Figure 10 shows the trained normal BPNN. The input of the normal BPNN is the RMS features of four-channel sEMG signals and the actual angle values *θ_knee_* denote the output. The numbers of neurons are 4, 12, and 1.

### 3.4. Comparison of Estimated Results

After three models were trained, we used the test set to evaluate the performance of the models. Firstly, we performed knee angle estimation using the adaptive regression model proposed in this paper. Secondly, to verify the improvement of our method, the DBN-BP model was used to perform the estimation task. In addition, a plain BPNN was used in the same work to provide additional comparisons.

**Figure 11:**
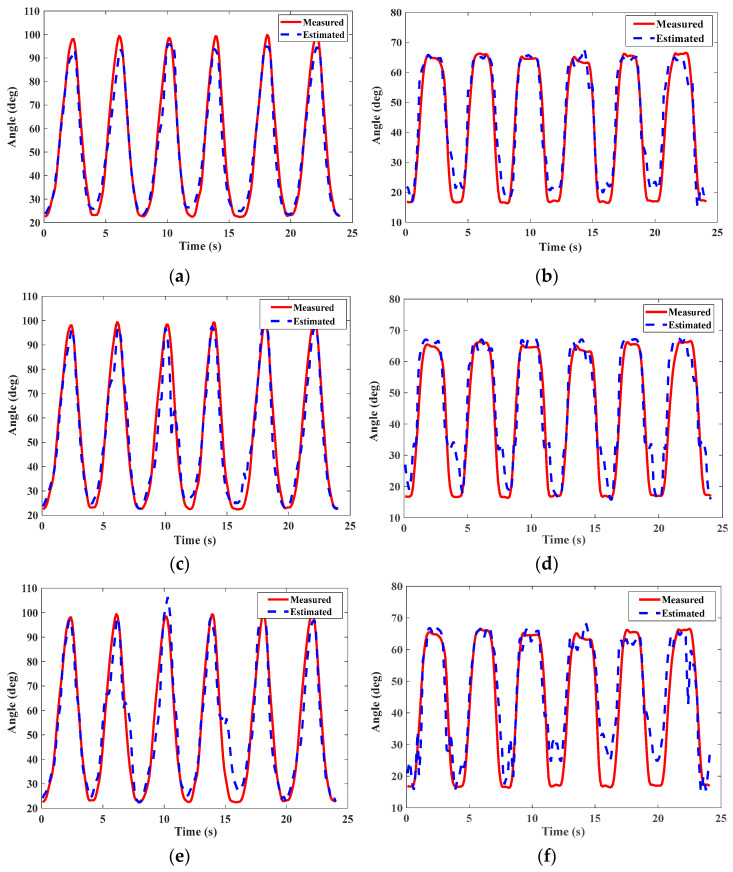
(**a**,**c**,**e**) Estimated results of the squat based on PSO-DBN-BP, DBN-BP, and BPNN. (**b**,**d**,**f**) Estimated results of the knee flex/ext based on PSO-DBN-BP, DBN-BP, and BPNN.

**Figure 12:**
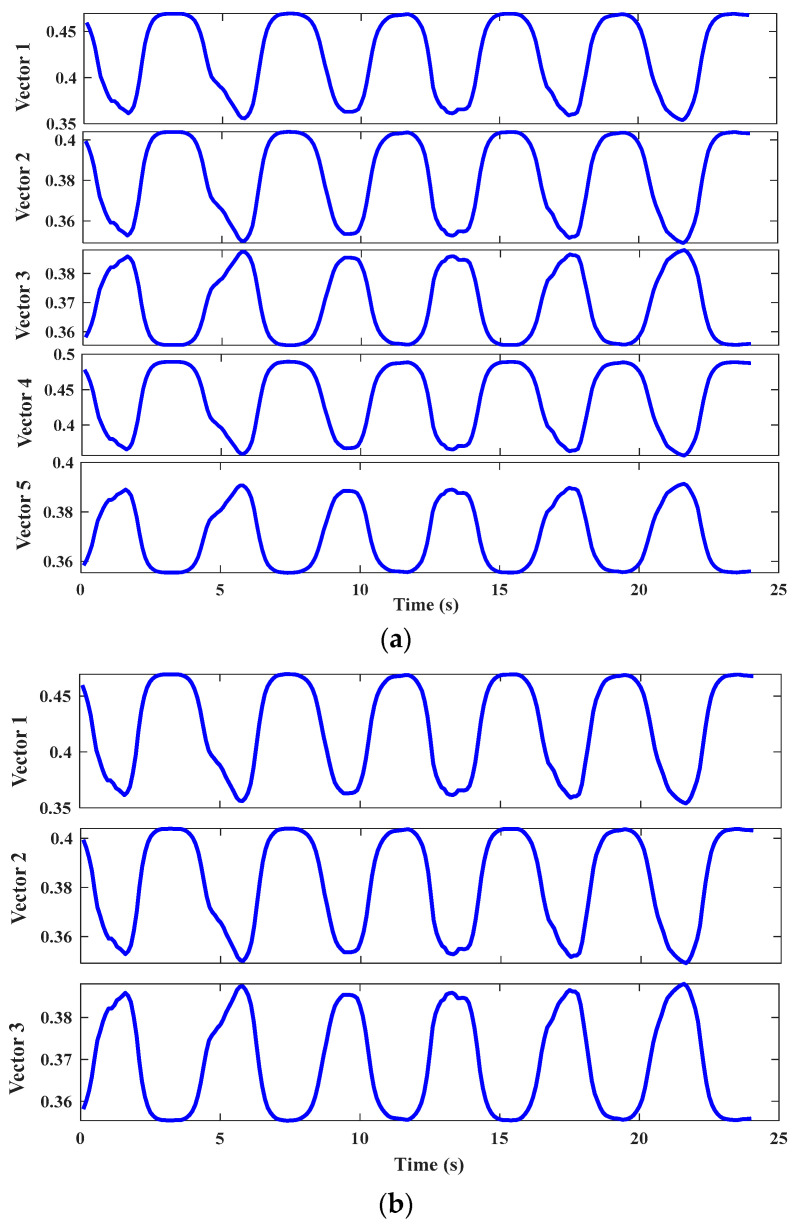
(**a**) The new five-dimensional reconstructed features for four-channel sEMG signals by using PSO-DBN. (**b**) The three-dimensional features by using DBN for dimensionality reduction.

Figure 11 reveals the estimation results of the three models of S1 using the test set samples. The Vicon measurement was taken as the “true” value. It could be seen from Figure 11c,d that the prediction result curves of DBN-BP could approximate the actual value in general, but there were more significant errors than our method. Such as 10 s, 15 s in Figure 11c and 5 s, 10 s, 15 s in Figure 11d. Compared with the DBN-BP model, our method achieved two improvements in the estimated results: (1) The estimated result was closer to the actual value. (2) The generated curve was smoother. These were more conducive to improving the accuracy and stability of the exoskeleton control system. The same test set was applied to the regular BPNN. As shown in Figure 11e,f, the BPNN model is also capable of estimating continuous knee joint motion based on sEMG signals. However, it is relatively difficult for this model to accurately estimate the joint angle, and DBN-BP outperforms BPNN as it can learn the high-level features of the original sEMG signals, as shown in Figure 12b.

In addition to the comparisons in Figure 11, we also quantitatively assessed the results. The trained models were applied to evaluate the test set of all subjects and calculated all the RMSEs, CCs, and R^2^ through (16), (17), and (18). Table 2 lists the means and standard deviations computed by fifteen tests. As can be seen in Table 2, our method presented significant advantages over the other two models. The most accurate results were obtained for squatting in S1 and knee flexion/extension in S3, representing the optimal performance achieved by our method under the same conditions. The average RMSEs of our method between the estimated results and the actual values were 9.42 ± 0.31° and 7.36 ± 0.25°, indicating that the error between the actual and estimated values was tiny. And the RMSEs of our method were significantly lower than 10.54 ± 1.16° (*p* = 0.001), 8.64 ± 0.61° (*p* < 0.005) of DBN-BP and 14.14 ± 1.56° (*p* < 10^−4^), 9.60 ± 0.86° (*p* < 10^−7^) of BPNN. In addition, the average CCs of our method were 0.96 ± 0.01 and 0.94 ± 0.01, which were numerically closer to 1. The average R^2^ scores are 0.92 ± 0.01 and 0.90 ± 0.01, indicating a good match between the curves generated by the estimation results and the measured curves. However, the CCs and R^2^ scores of the other two networks both indicated that their errors were much larger than our method.

## 4. Discussion

Accurate and real-time joint angle estimation is vital in human–robot interaction technology, and it is more valuable in the study of smooth and stable control of intelligent artificial limbs, exoskeletons, and rehabilitation robots than in motion classification. Xi et al. used the state space model based on HMM. However, this model involved the computation of many parameters, and the test time was only 10 s, so it should be tested in practice for a longer period of time [6]. Li et al. used a regression model based on MLP and a Savitzky–Golay filter to achieve a continuous motion estimation of knee and ankle joints [10]. This method took traditional time domain features as the input of the neural network. Traditional time domain features (MAV, RMS) present limited ability to the representation learning of sEMG signals and as a result, the robustness of the model is reduced. Due to the non-stationarity and individual differences of sEMG signals, the traditional fixed recognition model usually aims at specific subjects, which limits the generality of models. In these contexts, a basic commonality model based on adaptive deep belief network optimization was constructed in this paper. It satisfied the personalized demand for new subjects from the commonality model through the parameter update mechanism [1]. Compared with traditional methods, our method simultaneously considered and compensated for the impact of non-stationarity and individual differences in sEMG signals on estimation accuracy. The statistical results suggested that our method achieved consistent stability and high performance in different subjects.

In this study, the dimensionality of the optimal feature vectors reconstructed by PSO-DBN was higher than the initial feature vectors. As shown in Table 1, taking the squat of S1 as an example, the dimension of the features by the PSO-DBN was five. Our method was based on the feature dimensionality increase strategy, as opposed to the dimensionality reduction strategy used in [21]. Therefore, we compared the results of the two methods and found that the results based on high-dimensional features were better than dimensionality reduction features. However, the dimension reduction method could also perform well in some cases, such as the squatting of S4 and the flex/ext of S5, indicating that the dimension reduction method could also perform well at the higher signal-to-noise ratio signals. As shown in Figure 12b, the dimensionality reduction brought about information loss in the data, thus making it difficult to deal with complex data and consequently increasing the resulting error. However, our method projected sEMG sequences onto high-dimensional space and reconstructed sEMG features of different individuals in high-dimensional space. As shown in Figure 12a, the initial features were reconstructed effectively in high-dimensional space, enhancing the representation of sEMG features to learn more advanced information.

Moreover, increasing the dimension can lead to more optimized parameters, which can improve the fitting capacity of the network. Stable and accurate results were obtained among different subjects. In addition, increasing the feature dimension may help maintain relatively high accuracy when electrode shedding or electrode displacement occurs during the experiment so that the robustness of pattern recognition based on sEMG signals can be enhanced. In future research, we will focus on how to compensate for the lost data to meet the accuracy requirement when the electrode is accidentally detached.

Similarly, we have also selected a normal BPNN as another comparison method [9]. BPNN also requires stable voltage signals as input to achieve ideal results. However, since the BPNN model directly uses the RMS feature as input, it is the most significant of the three methods. As a result, satisfactory results cannot be obtained in most cases.

However, there are limitations to this study that require further elaboration before the findings can be applied to exoskeleton robot control. First, this study only collected the sEMG signals of a few healthy subjects. We are aware that there are differences between the characteristics of sEMG signals of subjects with lower limb disabilities and those of healthy subjects, which may affect the estimated results. Individual differences in subjects, such as gender, age, and body fat content, should be considered. Secondly, we selected only two motion modes related to knee joint motion, so additional motion modes should be considered to verify the effectiveness of our method. Finally, when subjects wear exoskeletons during the experiment, external loads and electrode displacement will also affect the results. Therefore, our method requires additional measures to compensate for the difficulties caused by the above limitations.

## 5. Conclusions

The non-stationarity and individual differences of sEMG signals greatly limit the generality of the traditional recognition models on new subjects. This paper proposed a knee joint continuous motion estimation method based on self-adaptive deep belief network optimization. An adaptive updating mechanism of model parameters was built to satisfy the personalized demand for new subjects. All the above methods were verified by collecting a dataset of five healthy subjects. The results indicated that the method proposed in this paper reduced the influence of non-linearity and individual differences of sEMG signals as compared to the traditional methods. Future research will continue to improve the model and highlight the continuous motion estimation of multi-joint angles in the lower limb. Complex locomotion patterns such as walking up/down stairs, running, and jumping will be considered. Human gait is coordinated and completed by multiple joints and muscles, so it is vital to explore the synergy of multiple muscles to improve the accuracy of the estimation results. At the same time, the estimated results will be adopted as a control instruction to realize the human–robot interaction (HRI) of the lower limb exoskeleton based on the sEMG signals.

## Figures and Tables

**Table 1:** Parameter optimization and training time results of PSO-DBN for all subjects.

Subject	Squat					Knee Flex/Ext				
	Number of Neurons in Each Layer				Time/s	Number of Neurons in Each Layer				Time/s
S1	37	26	16	5	31.7	8	48	11	44	40.4
S2	34	32	29	35	43.4	34	41	14	17	29.3
S3	2	41	48	32	36.6	38	2	42	49	42.0
S4	5	40	25	34	39.0	18	14	40	19	34.1
S5	2	49	46	42	41.1	17	28	37	32	41.4

**Table 2:** The average of the three evaluating indicators and standard deviations of all subjects.

Subject	Model	Squat			Knee Flex/Ext		
		RMSE	CC	R^2^	RMSE	CC	R^2^
1	PSO-DBN-BP	5.57 ± 0.44	0.99 ± 0.01	0.97 ± 0.01	8.71 ± 0.16	0.93 ± 0.01	0.88 ± 0.01
	DBN-BP	6.37 ± 1.04	0.98 ± 0.01	0.96 ± 0.01	9.09 ± 0.36	0.92 ± 0.01	0.85 ± 0.01
	BPNN	8.79 ± 2.71	0.94 ± 0.04	0.89 ± 0.07	9.84 ± 1.22	0.89 ± 0.03	0.79 ± 0.05
2	PSO-DBN-BP	9.96 ± 0.51	0.95 ± 0.01	0.90 ± 0.01	8.34 ± 0.17	0.93 ± 0.01	0.88 ± 0.01
	DBN-BP	11.72 ± 1.28	0.93 ± 0.01	0.86 ± 0.02	12.06 ± 0.41	0.90 ± 0.01	0.84 ± 0.02
	BPNN	12.90 ± 1.45	0.90 ± 0.01	0.81 ± 0.04	12.64 ± 0.47	0.88 ± 0.02	0.81 ± 0.02
3	PSO-DBN-BP	11.82 ± 0.10	0.95 ± 0.01	0.90 ± 0.01	6.09 ± 0.34	0.96 ± 0.01	0.93 ± 0.01
	DBN-BP	13.53 ± 2.61	0.93 ± 0.03	0.85 ± 0.06	7.21 ± 1.18	0.95 ± 0.02	0.89 ± 0.04
	BPNN	21.71 ± 1.40	0.81 ± 0.02	0.66 ± 0.03	8.77 ± 0.99	0.92 ± 0.02	0.85 ± 0.04
4	PSO-DBN-BP	10.49 ± 0.15	0.95 ± 0.01	0.90 ± 0.01	5.87 ± 0.34	0.97 ± 0.01	0.95 ± 0.01
	DBN-BP	10.86 ± 0.24	0.94 ± 0.01	0.89 ± 0.01	6.75 ± 1.30	0.95 ± 0.02	0.91 ± 0.04
	BPNN	12.32 ± 0.99	0.93 ± 0.01	0.87 ± 0.01	8.12 ± 0.86	0.93 ± 0.02	0.87 ± 0.03
5	PSO-DBN-BP	9.29 ± 0.33	0.96 ± 0.01	0.93 ± 0.01	7.77 ± 0.23	0.93 ± 0.01	0.86 ± 0.01
	DBN-BP	10.22 ± 0.62	0.95 ± 0.01	0.91 ± 0.02	8.07 ± 0.39	0.91 ± 0.01	0.84 ± 0.01
	BPNN	14.97 ± 1.24	0.88 ± 0.02	0.78 ± 0.04	8.64 ± 0.75	0.90 ± 0.02	0.82 ± 0.04
**Overall**	**PSO-DBN-BP**	**9.42 ± 0.31**	**0.96 ± 0.01**	**0.92 ± 0.01**	**7.36 ± 0.25**	**0.94 ± 0.01**	**0.90 ± 0.01**
	**DBN-BP**	**10.54 ± 1.16**	**0.95 ± 0.01**	**0.89 ± 0.02**	**8.64 ± 0.61**	**0.93 ± 0.01**	**0.87 ± 0.02**
	**BPNN**	**14.14 ± 1.56**	**0.89 ± 0.02**	**0.80 ± 0.04**	**9.60 ± 0.86**	**0.90 ± 0.02**	**0.83 ± 0.04**

## Data Availability

The datasets during the current study are not publicly available because of the further analysis of the datasets in our research. But they are available from the corresponding author upon reasonable request.
